# When legal rights are not a reality: do individuals know their rights and how can we tell?

**DOI:** 10.1080/09649069.2013.774764

**Published:** 2013-04-19

**Authors:** Catrina Denvir, Nigel J. Balmer, Pascoe Pleasence

**Affiliations:** Faculty of Laws, University College London

**Keywords:** legal aid, LASPO, self-help, self-representation, methodology

## Abstract

Public knowledge of rights has been the subject of a number of empirical enquiries over the last decade. In England and Wales, knowledge of rights and its relationship with an individual's capacity to ‘self-help’ and ‘self-represent’ when faced with a civil justice problem has become the subject of renewed attention following changes to legal aid which, from March 2013, will see the availability of legal advice and representation dramatically reduced. Previous studies focusing on public knowledge of rights in this (and other) jurisdictions have illustrated a lack of knowledge amongst the general population and more specifically, a widespread tendency of individuals to assume that the law aligns with their own moral, ethical or social attitudes. However, many of these studies have also suffered from methodological shortcomings. In attempting to address some of these shortcomings this study uses an open-ended format to ask individuals with one or one or more civil or social justice problems to describe their rights/legal position. We find that whilst an open-ended question approach to exploring knowledge of rights yields insight not acquired by other formats, its utility is constrained by difficulty reconciling articulation and actual knowledge of rights. We discuss the implications of these findings as they relate to the development of future research in the field of family and social welfare law, Public Legal Education (PLE) and access to justice post-March 2013.

## Introduction

In 2010 the British Government proposed wide-ranging reforms to the legal aid system in England and Wales (Ministry of Justice 2011). They noted that although ‘successive changes (had) managed to contain the growth in overall spending on legal aid, such changes (had) not addressed the underlying problems facing the scheme’ (Ministry of Justice 2010b, p. 3). In the long term, the Government agreed that simplification of the justice system to make its navigation easier for lay individuals was necessary; in the meantime, the availability of publicly funded specialist advice and representation across a range of matter types would be substantially curtailed (Ministry of Justice 2010a, 2010b, 2011). Deciding which areas of law would and would not qualify for publicly funded legal advice and assistance under the new legal aid scheme was justified in part, by reference to whether the public could utilise (self-help and) self-representation for their particular problem (Ministry of Justice 2010a, 2010b, 2011).^1^ Consequently, the reforms heralded by the Legal Aid, Sentencing and Punishment of Offenders Act (LASPO) 2012, detailed (explicitly and implicitly) that self-help/self-representation would from April 2013 onwards, become a key route to resolving civil justice problems.^2^

### Public legal capability, education and knowledge of rights

The idea that individuals are capable of handling civil justice problems without professional advice, anticipates a level of existing competence from the population – particularly those individuals who are neither eligible for legal aid, nor able to secure alternative legal services. How public legal capability is assessed and improved, or for that matter, the characteristics necessary to produce individual legal ‘capability’ remains an issue of some debate. One factor generally considered relevant, is the extent to which individuals know their legal rights (see, e.g., PLEAS Taskforce 2007, Gramatikov and Porter 2010, Buck *et al.*, 2008, Balmer *et al.*, 2010, Bowal 1999). It is argued that without such knowledge, individuals will more often fail to vindicate their rights (Bowal 1999); fail to take steps to protect themselves against the likelihood of a particular eventuality (Kim 1999, Meager *et al.*, 2002); fail to uphold their civic responsibilities (Bowal 1999); and experience diminished success when self-representing in court (Moorhead and Sefton 2005). A lack of knowledge has also been said to potentially generate unrealistic expectations of lawyers and judges (Bowal 1999) and the risk of experiencing a civil justice problem (Williams 2009) and is further linked with the adoption of poor problem handling techniques and lower levels of satisfaction with outcome when an individual seeks to handle their problem alone (Buck *et al.*, 2008, Balmer *et al.*, 2010, Denvir *et al.*, 2012).

In the Public Bill Committee debates regarding the passage of the LASPO Bill, it was said that changes to legal aid demanded greater public knowledge of rights if individuals were to be expected to self-help and self-represent more often (Public Bill Committee 2011). It follows, then, that as part of an on-going effort to safeguard access to justice, whether the general public have knowledge of their rights or not will remain one of the key issues facing public policy makers in the years to come. Studies which have previously sought to measure knowledge of rights have found that citizens in a number of jurisdictions frequently admit to lacking knowledge (see, e.g., Cortese 1966, Williams and Hall 1972, Saunders 1975, Baker and Emery 1993, Parle/IARS 2009, Casebourne *et al.* 2006; Genn *et al.*, 2006, Tennant *et al.* 2006, Albrecht and Green 1977) with this lack of knowledge more prominent among certain subsections of the population, namely young people, the mentally ill, and those without educational qualifications (see, e.g., Williams and Hall 1972, Ruck *et al.*, 1998, Youth Access 2002, Parle/IARS, 2009, Casebourne *et al.* 2006, Buck *et al.*, 2008, Denvir *et al.*, 2012). These concerns have been the basis for a range of policy initiatives aimed at improving public knowledge of rights, referred to as ‘Public Legal Education’ (PLE) (PLEAS Taskforce, 2007).^3^ Such interventions seek to rectify an absence of knowledge, and thus require an understanding of the existing level of knowledge amongst the general population. Yet measuring public knowledge of rights or for that matter the benefits that may flow from increased knowledge is no easy feat, as existing research in the field has illustrated.

### Measuring knowledge of rights

#### Self-reporting knowledge

Existing studies have generally taken one of two approaches in measuring public knowledge of rights. The first approach, as used by both Denvir *et al.*, (2012) and Buck *et al.*, (2008) has involved asking respondents to self-report the extent to which they know their rights, with results highlighting low levels of knowledge amongst the general population and certain groups. However, as recognised in the methodological literature (see, e.g., Baldwin 2009) whilst self-reporting is a convenient approach, it is not without issues. These include the fact that long reference periods in any type of survey may impact upon memory recall (Deming 1950, Bradburn *et al.*, 1987), that respondents to face-to-face surveys may be inclined to answer in certain ways (for a variety of reasons, see, e.g., Groves *et al.*, 2009, Calahan 1968), and that the acquisition of knowledge after the event may lead people to incorporate post-event information in their memory of the experience (Groves *et al.*, 2009). This last issue has particular relevance given that an individual's propensity to incorporate post-event information has ramifications for any attempt to link the presence of knowledge with the problem-resolution strategies individuals adopt. More problematic however, is the assumption that those who report knowing their ‘legal rights’ actually do. In the absence of an understanding of what respondents actually believe their rights to be, Buck *et al.*, (2008) and Denvir *et al.*, (2012) may simply indicate an individual's level of confidence in their knowledge, especially given that existing research also highlights individuals answer more confidently when asked fixed choice questions (see, e.g., Schuman and Presser 1981, Schwarz and Hippler 1991).

#### Fact-based questions with/without a contextual hypothetical scenario or ‘vignette’

Studies which have attempted to verify the self-reporting of knowledge such as Meager *et al.*'s (2000) employment rights research, have adopted a second approach. Using self-assessment questions alongside a number of other question types, including (prompted and unprompted) awareness of various employment related legislation, and questions which ask whether a hypothetical situation represents a breach of the law alongside requiring participants to name the law breached. Via these methods, Meager *et al.*, (2002) were able to demonstrate that although two-thirds of respondents to their study claimed to be well informed or very well informed about their rights, substantive knowledge varied by individual problem-type, with individuals often able to identify a breach of the law in a hypothetical situation, but unable to substantiate this breach by naming the relevant legislation.^4^ The authors concluded that individuals often identified breaches of the law based on perceptions of fairness or natural justice, rather than knowledge of the relevant legislation (Meager *et al.*, 2002) suggesting that self-reporting remained a crude proxy for actual knowledge.

Yet, whilst utilising a range of approaches, the authors failed to comment on the fact that the ability to name legislation was not necessarily a good measure of knowledge of rights or of the content of the legislation itself. By focusing more on whether respondents were able to link what they perceived to be unfair behaviour with the corresponding legislation, rather than exploring whether respondents actually understood their rights, the results provided only limited insight into ‘knowledge of rights'. Similar such limitations were evident in Kim's earlier (1999) work exploring US employees’ knowledge of rights regarding at-will-dismissal. Respondents were required to indicate whether they felt the dismissal was lawful or unlawful in respect of a number of circumstances. But whilst incorrect responses were said to indicate a confusion of legal norms and ethical or social norms, correct responses were deemed to indicate ‘knowledge’ of rights, rather, than as may well have been the case, a situation in which ethical/social norms and the law happened to align (Kim 1999) or ‘educated’ or ‘wild’ guesses happened to be correct (Nadaeu and Niemi 1995).^5,6^

A different approach to exploring public knowledge of rights (whilst retaining a fact-based questioning approach) was utilised a few years later by Barlow *et al.* in 2005. With the intent of exploring existing levels of knowledge among the general population as to the rights of a cohabitant vis à vis the rights of a spouse,^7^ the authors developed a series of fact-based questions relating to the legal rights of a protagonist in a hypothetical scenario (or ‘vignette’) which were then added to the 2004 British Social Attitudes Survey (BSAS). Finding that individuals held erroneous beliefs in respect of time-dependent accrual of benefits^8^ and a belief in the equivalence of paternal rights and responsibilities between married and unmarried couples, Barlow *et al.* (2005, p. 45) concluded that people's beliefs about cohabitation law, whether accurate or not, were based less on acquired knowledge, and more upon ‘notions of social logic, fairness and morality’.

Whilst findings corroborated those of other related studies (e.g. Meager *et al.*, 2002, Kim 1999) as Pleasence and Balmer (2012) observed, the BSAS questions relied on a presumption that people had good knowledge of the spousal rights against which cohabitants’ rights were being compared, resulting in conflation between knowledge of spousal rights and knowledge of cohabiting rights. Thus, in their 2012 study on the same topic using respondents to the English and Welsh Civil and Social Justice Panel Survey (CSJPS), Pleasence and Balmer (2012) also developed a series of fact-based questions in relation to a hypothetical scenario but randomly varied the duration of the relationship (one month to 20 years – allowing formal modelling of time dependency in beliefs about accrual of rights) and its status (cohabiting or married). Whilst employing a different methodology, like Barlow *et al.*, (2005), Pleasence and Balmer (2012) found evidence of ongoing public misconception of cohabitation law, leading the authors to propose that in the absence of an actual understanding of the law, the public tended to believe the law aligned with the rights they thought it ought to protect.

Although Pleasence and Balmer (2012) were able to address some of the issues evident in the ‘vignette’ approach taken by Barlow *et al.*, (2005) this does not leave the approach limitation-free. Other signs point to the fact that responses to hypothetical questions may, like self-reporting, provide only a rough measure of knowledge for a number of reasons. Nadaeu and Niemi (1995) note that some individuals, even when unsure of an answer, will be compelled to respond in a manner other than ‘don't know’ with some people inclined to answer knowledge-based questions even in the face of considerable uncertainty (Chong 1993). For some, these uncertain answers are ‘educated guesses', for others they may be ‘wild guesses’ (Nadaeu and Niemi 1995). In addition, it has been said that most people ‘construct’ attitudes when asked a question (Tourangeau and Rasinski 1988), meaning that a respondent without actual knowledge may answer a fact-based question in manner no different to how they would answer an attitudinal question; perhaps explaining why previous research has found that individuals respond with answers indicative of what they think the law *should* be, rather than what it actually is. The question that these issues highlight, is whether there might be an alternative approach to exploring public knowledge of rights, and if so, what form it would take?

#### Alternative approaches to exploring public knowledge of rights

In the exploration of public knowledge of rights, open-ended questions appear to have remained if not unused, then seemingly unreported. This is somewhat surprising since as Hruschka *et al.*, (2004) and Geer (1988) note, the use of open-ended questions offers a number of benefits, including capturing data of (potentially) greater accuracy or relevance to the individual, alongside capturing unanticipated responses. What may offset these benefits (and account for the lack of use to date) is as both Griffith *et al.* (1999) and Johnson *et al.*, (1974) note (although contested by Burchall and Marsh 1992) these benefits may come at the expense of higher response rates, possibly because some individuals (more often those without educational qualifications according to Geer 1988) struggle to articulate their responses.

However, neither of these suggested limitations would appear to outweigh the potential insight open-ended questions might provide when used to explore how the public defines and describes their rights. This is particularly so in respect of knowledge of the law, since self-help and self-representation rely (to varying degrees) on an individual's ability to identify and articulate an infringement of their rights. In the context of wide-ranging changes to public legal aid in England and Wales and a presumed increase in self-help and self-representation (Public Bill Committee 2011), open-ended questions present a fresh approach to the measurement of public knowledge of rights across a range of civil and social justice issues.

Accordingly, in this study we explore how individuals with one or more civil or social justice problems respond when asked to briefly describe their rights/legal position in the context of a quantitative survey. Our findings contribute to the existing methodological and PLE literature by: (a) examining the merits of using open-ended questions to explore public knowledge of rights; and (b) exploring how the public articulate their rights when asked to do so and what may be inferred about their level of knowledge. With the changes contained in the Legal Aid, Sentencing and Punishment of Offenders Act 2012 (LASPO) imminent, this study is particularly timely.

## Aims and hypothesis

In this study, we draw on data from the 2010 English and Welsh Civil and Social Justice Panel Survey (CSJPS) to build upon earlier studies exploring public knowledge of rights. Utilising self-assessment and open-ended (verbatim) responses from the CSJPS, this study sets out to determine: whether an open-ended question format can provide useful insight into an individual's knowledge of their rights and/or the validity of their self-assessment and the extent to which findings might provide useful lessons for research and policy development.

Referring to self-assessed knowledge of rights and on the basis of the issues raised in the extant literature (including the findings of Williams and Hall 1972, Caseborne *et al.* 2006, Genn *et al.*, 2006, Tennant *et al.* 2006, Buck *et al.*, 2008 and Denvir *et al.*, 2012) we hypothesise that the majority of those experiencing one or more civil or social justice problems do not report having knowledge of their rights and that the majority report a failure to acquire knowledge of their rights, but as found by Kim (1999) and Meager *et al.*, (2002) these rates will vary by problem-type. Based on Deming's (1950) concerns regarding issues of memory recall in relation to self-assessed questions, we also hypothesise that those who self-report report knowing their rights in Wave 1 of the CSJPS in respect of a particular problem, frequently answer inconsistently when they are asked whether they knew their rights in Wave 2 (in relation to the same problem). We further hypothesise that many of the people who profess to have knowledge of rights are unable to articulate these rights in response to the open-ended format, however unlike Geer (1988) we propose that the failure to articulate knowledge of rights will not be associated with education level.

## Methods

### Data

Data used for this study came from the 2010 CSJPS,^9^ a nationally representative survey of 3806 individuals aged 16 or over, living in 2318 households across 194 sample points in England and Wales. The survey explored respondents’ experience of, and response to a broad range of problems involving legal rights. The survey was conducted face-to-face in respondents’ homes, with all members of households interviewed separately. The survey averaged 37 minutes, with a household response rate of 88% and a cumulative eligible adult response rate of 54%. For further technical details of the CSJPS, see Pleasence *et al.*, (2011). Table 1 shows incidence of each of the broad problem types in the survey

**Table 1.** Prevalence of civil justice problems of different types.

Problem type	N	%
Neighbours	359	9.3
Consumer	338	8.9
Employment	211	5.5
Money	202	5.3
Debt	185	4.9
Benefits	166	4.4
Personal Injury	155	4.1
Rented housing	144	3.8
Relationship breakdown	80	2.1
Education	71	1.9
Owned housing	59	1.6
Clinical negligence	53	1.4
Divorce	41	1.1
Domestic violence	39	1.0
Care proceedings	9	0.2

In addressing some of the limitations of earlier research, the 2010 CSJPS included a series of open-ended questions, including questions designed to ascertain if individuals knew their rights/legal position. It did so by asking individuals who reported one or more civil or social justice problem the following: ‘Thinking about the time the problem first started, to what extent did [you/your partner] understand [your/their] legal position – for example, what [your/their] legal rights were?’ Which had a scale of answers as follows: (1) completely; (2) mostly; (3) partly; (4) not at all; (5) don't know. Those who answered with (3), (4) or (5) were then asked ‘To what extent do [you/they] now understand what [your/their] legal position was?’ This differed from the wording of the continuous Civil and Social Justice Survey (upon which Buck *et al.*, 2008 and Denvir *et al.*, 2012 relied) which asked ‘At the time of the [problem descriptor], did you know what your legal rights were relating to this problem?’ with the intent of negating some of the memory recall problems (Deming 1950, Groves 2009) mentioned above by distinguishing between those who knew their rights from the offset and those who acquired knowledge. Those who stated that they knew their rights at the start of the problem ‘completely’ or ‘mostly’ and those who indicated that they now knew their rights ‘completely’ or ‘mostly’ were additionally asked whether they ‘[could] describe, briefly, what [your/your partner's] legal position was?’

In Wave 2 of the CSJPS, all follow-up respondents were asked again ‘To what extent do [you/they] now understand what [your/their] legal position was?’ in respect of the same problem, with the same range of responses available ('completely', ‘mostly', ‘partly', ‘not at all', ‘don't know'). This enabled the tracking of the self-assessment respondents gave, across CSJPS waves. However, owing to timing restrictions, follow-up respondents in Wave 2 were not asked to briefly describe their rights, so where an individual responded that they knew their rights ‘partly', ‘not at all’ or ‘don't know’ in Wave 1, but in Wave 2 indicated that they now knew their rights, the verbatim responses of these individuals were not captured.

### Analysis

Firstly we set out the extent to which respondents to the 2010 CSJPS (Wave 1) self-report knowledge of rights and how this varies with problem-type so as to provide an overview of the cohort and a basis for comparison with existing studies. We describe the rate at which individuals knew their rights at the start of their problem and the rate at which individuals acquired knowledge of their rights, distinguished by problem-type.

Exploring how self-reported knowledge changes over the course of the reference period, using descriptive statistics, we compare individuals’ self-reported knowledge in Wave 1, against their self-reported knowledge in Wave 2, looking also at the extent to which knowledge varies by problem-type across Wave 1 and Wave 2 of the CSJPS.

Finally, we look at how individuals answered the open-ended question asking them to describe their rights. We code these verbatim responses into one of seven categories as outlined below. Whilst responses were not ‘marked’ as correct or incorrect, we anticipated that those inferring the intended meaning from the question and capable of articulating their legal position/rights, would provide an answer that gave insight into their position of (legal) power relative to the other party. For example, someone who purchased a faulty coat and reported a consumer problem could (in an ideal scenario) answer the question as follows: (a) ‘under the Sale of Goods Act I was allowed to claim a refund or exchange on my faulty coat'. Those without knowledge of or reference to the legislation might have offered a similar explanation such as: (b) ‘the store had to provide me with a refund or exchange'. In consideration of the fact that in the context of a long quantitative survey some participants might have (reasonably) opted to explain their legal position with greater brevity (see further Herzog and Bachman 1981), we could also have expected answers such as (c) ‘I could get a refund’ which although shorter, remained illustrative of an individual's knowledge of their rights/legal position.

Whilst the range of expected answers was rather broad, individuals actually answered in a far more varied manner than this. Analysis of all verbatim responses identified seven different responses types that individuals tended towards in answering the question. These categories included:

(1) Those who described their situation or the outcome they experienced (e.g. ‘My zipper was broken on my coat’ or ‘The shop offered a refund');
(2) Those who referred to legislation, used legal terminology, spoke of ‘positive’ rights, or provided a common sense interpretation of their rights (e.g. see (a), (b) and (c) above);
(3) Those who referred to legislation, used legal terminology, or spoke of ‘positive’ rights but whose response indicated that they may have misinterpreted their legal position, or over/underestimated their rights (e.g. an example of an overestimate of rights might have included: ‘Under the Sale of Goods Act my rights were to get a new coat and the shop had to replace it with a better quality brand than previously and compensate me for inconvenience caused'. Whereas an underestimation of rights might have been: ‘I was entitled to a refund or return but it was the manufacturer I needed to contact not the store');
(4) Those who made a value judgement about the fairness of the legal system, the individuals involved, or their position in it (e.g. ‘I was right, the coat was faulty’ or ‘No point in complaining, the Government is acting to take away the rights of consumers');
(5) Those who referred to broad rights that did not suggest any knowledge of their rights in relation to their problem, but were indicative of a more general understanding of the legal system or with moral or ethical interpretations of the concept ‘right’ (e.g. ‘Human Rights', ‘The right to voice my opinion', ‘I could appeal', ‘I had the right to be listened to');
(6) Those who claimed to have no legal position/rights or to be ‘in the wrong’ (e.g. ‘No rights', ‘I should have noticed the zip was broken before I took the coat home, it was my fault I didn't check');
(7) Those who claimed not to know, or who failed to articulate anything.

Whilst the answers provided were for the most part discrete, where an individual provided a response that could have fallen into two categories, they were assigned to a category based on the length of their answer.^10^ This was not the case where an individual provided a response that may have fallen into category (2) and another category, they were coded only as (2) since the central issue was whether they could explain their legal/position rights, and not the information they provided surplus to this.

In exploring open-ended responses, we compare verbatim response-type against problem type, looking also at how individuals verbalised their rights, before turning to explore whether response and articulation type varied by level of educational attainment.

## Results

### Perceived knowledge and the acquisition of perceived knowledge of rights

Out of 1760 problems, respondents said they knew their rights at the outset of the problem, ‘completely’ for 396 problems (22.5%), ‘mostly’ for 319 problems (18.1%), ‘partly’ for 356 problems (20.2%), ‘not at all’ for 612 problems (34.8%). For a further 68 problems (3.9%)^11^ respondents suggested that they did not know whether or not they knew their rights. For those 1036 problems where the respondent answered ‘partly', ‘not at all’ or ‘don't know', they were additionally asked whether they later came to understand their rights. For 140 problems (13.6%) respondents claimed to have acquired knowledge of their rights ‘completely’ in relation to the problem, for 205 problems respondents acquired knowledge of their rights ‘mostly’ (19.9%), ‘partially’ for 345 problems (33.5%), for 289 problems (28.1%) respondents claimed still to have obtained no knowledge and for 51 problems respondents claimed not to know (5%).

In total, discounting those who provided no response, for 715 problems (41%) respondents claimed to know their rights completely or mostly at the start of the problem. For a further 345 problems (20%) respondents came to acquire knowledge of their rights, and for 685 problems (39%), respondents did not come to acquire knowledge of their rights. Table 2 identifies these individuals on the basis of problem type.

**Table 2.** Problem types and knowledge of rights.

	Knew rights at outset		Acquired knowledge		Did not acquire knowledge	
Problem type	*N*	*Row* %	*N*	*Row* %	*N*	*Row* %
Consumer	174	58.4	38	12.8	86	28.9
Employment	69	35.0	47	23.9	81	41.1
Neighbours	103	40.9	33	13.1	116	46.0
Owned housing	26	49.1	15	28.3	12	22.6
Rented housing	41	34.5	24	20.2	54	45.4
Debt	61	36.1	39	23.1	69	40.8
Money	72	39.3	38	20.8	73	39.9
Benefits	43	33.0	28	21.7	58	45.0
Education	22	33.8	11	16.9	32	49.2
Personal injury	29	42.6	17	25.0	22	32.4
Clinical negligence	19	39.6	5	10.4	24	50.0
Divorce/relationship breakdown	44	37.0	35	29.4	40	33.6
Domestic violence	10	27.0	13	35.1	14	37.8
Care proceedings	2	25.0	2	25.0	4	50.0

As is shown in Table 2, those with consumer problems more often knew their rights from the outset at 58.4% but were relatively less likely to acquire knowledge of their rights, followed by owned housing at 49.1% where the acquisition of knowledge was higher than non-acquisition. In domestic violence cases the acquisition of knowledge was higher than its rate at the outset, but those with domestic violence problems were more likely still, to not acquire knowledge at all, at a rate of 37.8%. A failure to acquire knowledge was particularly prevalent for care proceedings, clinical negligence, education and neighbours cases.^12^

### Self-reported knowledge across CSJPS waves

Owing to the panel format of the CSJPS, individuals at Wave 1 were asked whether they knew their rights at the start of the problem or whether they later came to acquire knowledge. In Wave 2, follow-up respondents (i.e. the same respondents as Wave 1) were again asked whether they ‘now knew’ their rights in relation to the same problem, allowing an exploration of whether respondents changed their self-assessment over time. Results found that 92 respondents (24.6%) claimed to have lost knowledge – that is to say they reported knowing their rights to a higher degree in Wave 1, than they did in Wave 2. 135 respondents (36.1%) claimed the same level of knowledge, and 147 respondents (39.3%) claimed to have gained knowledge. Table 3 displays the rate at which knowledge was lost, gained or remained the same by problem type.

**Table 3.** Whether respondents lost, gained or retained the same level of knowledge of their rights by problem-type.*

	Lost		Same		Gained	
	*N*	*Row* %	*N*	*Row* %	*N*	*Row* %
Consumer	6	17.6	20	58.8	8	23.5
Employment	10	21.7	15	32.6	21	45.7
Neighbours	20	25.6	18	23.1	40	51.3
Owned housing	1	7.1	10	71.4	3	21.4
Rented housing	9	34.6	10	38.5	7	26.9
Debt	9	21.4	17	40.5	16	38.1
Money	11	30.6	13	36.1	12	33.3
Benefits	5	23.8	8	38.1	8	38.1
Education	3	27.3	3	27.3	5	45.5
Personal injury	4	33.3	2	16.7	6	50.0
Clinical negligence	2	18.2	4	36.4	5	45.5
Divorce/relationship breakdown	10	31.3	10	31.3	12	37.5
Domestic violence	2	20	4	40	4	40.0

* With only one respondent the category ‘care’ was removed from the table, this respondent reported that their knowledge had stayed the same.

As is shown in Table 3, those with neighbours (51.3%), personal injury (50%), employment (45.7%), clinical negligence (45.5%), and education (45.5%) problems were more likely to report gains in knowledge than those with other problem types. Those with owned housing (71.4%) and consumer (58.8%) were most likely to report the same level of knowledge across waves, with those with rented housing and personal injury problems reporting a loss in knowledge more frequently than other problems types. However, at a rate of 34.6% for rented housing and 33.3% for personal injury, approximately two-thirds of individuals with these sorts of problems reported the same, or a gain in knowledge. For all problem types, more individuals reported either gaining or retaining the same level of knowledge than reported losing knowledge.

### Open-ended articulation of rights

For the 1056 problems where individuals claimed to know their legal position/rights ‘completely’ or ‘mostly’ at either the outset of the problem or later during the course of the problem, individuals were asked to briefly explain their legal position/legal rights. Analysis of the responses revealed some common themes categorised as described previously.

Table 4 examines the categorisation of verbatim responses in relation to problem-type.

**Table 4.** Categorisation of verbatim responses against problem-type.

	(1)		(2)		(3)		(4)		(5)		(6)		(7)	
	Situation/Outcome		Rights/Legal Position		Potential Error		Value Judgement		Vague Rights		No Legal Position/Rights		Don't Know	
	*N*	*Row* %	*N*	*Row* %	*N*	*Row* %	*N*	*Row* %	*N*	*Row* %	*N*	*Row* %	*N*	*Row* %
Consumer	46	21.7	111	52.4	12	5.7	5	2.4	14	6.6	7	3.3	17	8.0
Employment	32	27.6	36	31.0	7	6.0	9	7.8	11	9.5	11	9.5	10	8.6
Neighbours	33	24.3	41	30.1	2	1.5	17	12.5	20	14.7	8	5.9	15	11.0
Owned housing	12	29.3	13	31.7	0	0.0	3	7.3	6	14.6	1	2.4	6	14.6
Rented housing	17	26.2	32	49.2	3	4.6	1	1.5	4	6.2	0	0.0	8	12.3
Debt	29	29.0	46	46.0	0	0.0	2	2.0	2	2.0	3	3.0	18	18.0
Money	39	35.5	43	39.1	0	0.0	6	5.5	8	7.3	5	4.5	9	8.2
Benefits	14	19.7	34	47.9	0	0.0	6	8.5	0	0.0	4	5.6	13	18.3
Education	4	12.1	15	45.5	2	6.1	2	6.1	1	3.0	3	9.1	6	18.2
Personal injury	12	26	17	37.0	0	0.0	9	19.6	4	8.7	1	2.2	3	6.5
Clinical negligence	7	29.2	6	25.0	0	0.0	1	4.2	6	25.0	1	4.2	3	12.5
Divorce/relationship breakdown	23	29.5	36	46.2	1	1.3	3	3.8	3	3.8	3	3.8	9	11.5
Domestic violence	4	20.0	13	65.0	0	0.0	0	0.0	3	15.0	0	0.0	0	0.0
Care	2	50.0	0	0.0	0	0.0	0	0.0	2	50.0	0	0.0	0	0.0
Total	274	25.9	443	42.0	27	2.6	64	6.1	84	8.0	47	4.5	117	11.1

As can be seen, 25.9% of verbatim responses were categorised as instances where the respondent described the situation or outcome of the dispute. Forty-two per cent of respondents detailed their legal position/rights referring to the legislation, legal terminology or presenting a lay interpretation and fewer (8%) individuals provided a more general/vague interpretation of their rights. Less frequently, individuals gave responses which suggested they may have under or overestimated their rights or the strength of their legal position at a rate of 2.6%, with 4.5% claiming that they had no rights in respect of their problem or that their ‘legal’ position was that they were to blame or at fault. 6.1% made a value judgement about the legal system in response to the question, with a further 11.1% claiming that they did not know or could not verbalise their rights/legal position when asked.

Setting aside the categorisation of verbatim responses related to care proceedings given the small numbers contained within that group (four in total) results further highlight how individuals with certain problems tended to reply in certain ways. This was not so evident in respect of those who responded to the open-ended question in a manner that described their situation or outcome. Here, relatively high rates of responding in this manner were seen across most problem types and differences were relatively small with the exception of those with education problems (at 12.1%). Where respondents did describe their situation or outcome, their answers provided relatively little if any insight in to what they believed their rights to be, making it difficult to deduce whether they had any clear understanding of their rights as they believed they did. Examples of those who claimed they knew their legal position/rights included: ‘Took out a joint mortgage with a friend who had unknown to me a poor credit rating’ and ‘The agency gave me a poor score I had not done anything wrong they would not let me see my score in details'. Individuals with debt problems frequently spoke of the nature of their problem, rather than articulating what their legal entitlements were. Examples included one who simply stated that they ‘went over their overdraft’ and another who explained that ‘I gave girlfriend money to look after she spent it all.’

In respect of those who did answer the question in the manner expected by describing their rights/legal position, these types of responses were most common among those experiencing domestic violence problems at a rate of 65%. Responses such as ‘I could get him nicked’ or ‘I had the right to press charges’ were typical of domestic violence cases. Those with consumer, rented housing, benefits, divorce/relationship breakdown, debt and education problems also had high rates of providing answers that indicated that they had an understanding of their legal position/rights at a rate of between 52–45%. However, whilst responses were indicative of some level of knowledge, the extent to which respondents were familiar with the legislation varied from individual to individual. For example, some respondents provided relatively simplistic and short responses, such as ‘had to pay the money'. Whilst this response presents an efficient explanation in the context of a long quantitative survey it is distinguished from those who demonstrated willingness to provide a more detailed response, by stating their obligations, along with their entitlements, e.g. ‘had to repay the money in affordable instalments’ or ‘I had to pay the money back at a payment that was affordable to me and not what they were asking'. Some individuals gave specific reference to the relevant legislation in their answers with this more common in the case of those reporting clinical negligence or consumer problems. For example, whilst some responses were again simplistic, e.g. ‘could get a refund', others were more sophisticated, e.g. ‘goods not sold as described so they did not meet Sale of Goods Act', with another detailing that they had a ‘right to receive goods fit for purpose'. Although, individuals did not always recall the name of the legislation correctly, for example, one respondent claimed to be ‘entitled to a replacement under the (non-existent) faulty goods act’ this error did not compromise their understanding of their rights – namely that they were entitled to a replacement.

Yet, although frequently reporting their rights/legal position as expected, those with consumer problems along with those experiencing employment, rented housing and education problems were also more frequently categorised as responding to the open-ended question in a manner that indicated they may have erred in their understanding of their rights, along with either potentially over or underestimating their entitlements (5.7%, 6%, 4.6%, 6.1% respectively). Generally, this overestimation appeared to derive from a misunderstanding of the relevant legislation or its application. Some errors had the potential to impact upon the resolution of the problem and an individual's satisfaction with this resolution. For example, one respondent claimed to have ‘had the right to (have his vehicle repaired) to (his) satisfaction’ when in fact the relevant legislation (Sale of Goods Act 1979) imposes an objective test of satisfactory quality (under s14 (2) and s48B as it relates to repair) suggesting the individual may have been waiting for an outcome which holds the other party to a higher standard than the legislation does. There also appeared to be confusion surrounding tenancy rights, including one individual who erroneously believed it was within their rights to ‘withdraw rent until (their) property (was) sorted', a position which is not only legally incorrect, but places the individual at risk of eviction. Conversely, individuals also appeared to occasionally underestimate their rights, including one individual who claimed that ‘if you order something and pay on the Internet and tick the terms and conditions you have no rights’ a position at odds with the entitlements enshrined in the Consumer Protection (Distance Selling Regulations) 2000 and potentially also the Unfair Terms in Consumer Contracts Regulations 1999.^13^ There was indication of potential underestimation of rights in respect of employment, with a number of individuals claiming that they had to put up with changes to the terms and conditions of their employment or get another job. Whilst understanding that they did not have to agree to such changes, they did not appear to have an appreciation of the fact that (legally speaking) getting another job was not their only alternative.

Value judgements were most common amongst those with neighbours (12.5%) and those with personal injury (19.6%) problems. Typically these were the types of problems where individuals provided very matter-of-fact and short responses to the question asked by detailing that they were ‘in the right', ‘wholly innocent’ or ‘had done nothing wrong'. Whilst these were legitimate responses to the question asked, particularly since the question asked individuals to detail their ‘legal position', they did not elicit the desired information, namely greater insight into what people thought their rights were. It was also somewhat surprising to find responses discussing issues such as guilt/innocence in relation to a civil, rather than a criminal justice problem. However, this is perhaps less surprising given that personal injury problems tend to focus on the attribution of blame and responsibility, whilst neighbours problems can often straddle both the civil and criminal law.

In a similar vein, those with employment and education problems more frequently gave answers indicative of them having ‘no rights’ with one individual claiming that they had no rights because they ‘(could not) argue against council or government decision'. At a rate of 5.9% and 5.6% those with neighbours and benefits problems also expressed their belief that they had ‘no rights’ when asked. However, whilst some appeared to be more of a political statement than an expression of legal understanding, for example one individual in respect of a benefit problem who claimed that the ‘government acting (was) to remove rights', other responses appeared illustrative, not of knowledge (or a lack thereof) but whether a right could reasonably be upheld by the individual. For example, one respondent with a neighbour problem claimed that ‘essentially we had little rights, collecting evidence would be difficult and time consuming and we have to prove damage to property and then start civil action'. Similar such statements were evident in relation to employment problems where individuals often claimed that they had ‘no rights', not because none existed, but more often because they believed exercising such rights would put their job at risk.

Common sense interpretations of rights were most often given in respect of clinical negligence, domestic violence, neighbours and owned housing. Examples included those who identified a ‘right to take an individual to court', ‘the right to an appeal', ‘human rights', or the ‘right to be heard’ or ‘voice an opinion'. Whilst these responses were again, not incorrect, they were indicative of a more general understanding of an individual's position based on common sense or principles of fairness, one not reliant on knowledge of the law in relation to a particular problem. Of course many people may also have had knowledge of the law in respect of the question asked –indeed one individual did make a distinction between what she saw as legal and moral rights in stating in respect of her employment problem that she ‘Did not have legal right to move but had moral rights not to be moved further away from home'. However, because the rights these individuals professed to have were so generic, it made it difficult to determine the extent to which these individuals had any clear understanding of the specific rights relative to their legal problem, or whether they were simply relying on a broad understanding of concepts of ‘natural justice’ or legal common sense.

Importantly, verbatim responses highlighted a disjuncture between an individual's belief that they knew their rights/legal position and their ability to articulate this. This was more so the case for those reporting debt, benefits, education and owned housing problems than those reporting other problem types. For 117 problems (11.1%) individuals responded that they ‘(didn't) know’ when asked to articulate their rights, suggesting that they were confident enough to believe that their problem engaged a right but they could not explain this in legal terms. Indeed, one respondent said as much, claiming that they ‘(couldn't) really describe it’.

### Education level and responses to the verbatim question

Table 5 shows the categorisation of verbatim responses in relation to the level of educational attainment of the respondents. Whilst there are some minor differences apparent in Table 5 as shown below, there was no significant relationship emerging between type of verbatim response and level of education qualification. ^14^

**Table 5.** Categorisation of verbatim responses in relation to level of education attainment of respondent.

	(1)		(2)		(3)		(4)		(5)		(6)		(7)	
	Situation/Outcome		Rights/ Legal position		Potential Error		Value Judgement		Vague Rights		No Legal Position/Rights		Don't Know	
	*N*	*Row* %	*N*	*Row* %	*N*	*Row* %	*N*	*Row* %	*N*	*Row* %	*N*	*Row* %	*N*	*Row* %
None	41	24.1	66	38.8	3	1.8	10	5.9	19	11.2	6	3.5	25	14.7
Higher degrees	21	24.7	34	40.0	3	3.5	5	5.9	4	4.7	6	7.1	12	14.1
First degrees or equiv.	63	29.4	97	45.3	5	2.3	7	3.3	13	6.1	6	2.8	23	10.7
Diplomas in HE or equiv.	35	27.3	48	37.5	5	3.9	11	8.6	10	7.8	6	4.7	13	10.2
A/AS levels or equiv.	40	27.4	55	37.7	6	4.1	9	6.2	15	10.3	9	6.2	12	8.2
GCSE A-C or equiv.	15	23.4	35	54.7	1	1.6	3	4.7	2	3.1	2	3.1	6	9.4
GCSE D-G or equiv.	13	24.1	21	38.9	1	1.9	5	9.3	6	11.1	2	3.7	6	11.1
Trade apprentice	44	23.7	85	45.7	3	1.6	13	7.0	13	7.0	10	5.4	18	9.7
Other	2	22.2	2	22.2	0	0.0	1	11.1	2	22.2	0	0.0	2	22.2

## Discussion

Our results indicate that most individuals felt unsure of their rights at the outset of their problem. For 41% of problems, respondents claimed to know their rights completely or mostly at the start of the problem, for a further 20% of problems respondents acquired knowledge, and for 39% of problems respondents did not come to acquire knowledge.

Knowledge varied by problem type (see Table 2), with those with consumer problems more often reporting knowledge of their rights from the outset at 58.4% but relatively less likely to acquire knowledge of their rights, followed by owned housing at 49.1 % where the acquisition of knowledge was higher than non-acquisition. In domestic violence cases the acquisition of knowledge was higher than its rate at the outset, but those with domestic violence problems were more likely still, to not acquire knowledge at all, at a rate of 37.8%. A failure to acquire knowledge was particularly prevalent for clinical negligence, education and neighbour problems.

Utilising data from both Waves of the CSJPS highlighted that 24.6% of respondents claimed to lose knowledge with 39.3% claiming a gain in knowledge. As is shown in Table 3, those with neighbours (45.7%), personal injury (50%), employment (45.7%), clinical negligence (45.5%), and education (45.5%) problems were more likely to report gains in knowledge than those with other problem types. Those with owned housing (71.4%) and consumer (58.8%) problems were most likely to report the same level of knowledge across waves. Those with rented housing (34.6%) and personal injury (33.3%) problems reported a loss in knowledge more frequently than those with other problem types.

Analysis of verbatim responses demonstrated a number of response types, most common of these were responses in which individuals were able to give a brief overview of their rights/legal position. However, response types varied from those who supported their answer with reference to the relevant legislation to those who simply stated their rights. In respect of this latter group, although their answer appeared correct in relation to their dispute, it was not possible to deduce the basis of their knowledge, i.e. whether it stemmed from an understanding of the legislation, or whether it was based on a common sense position as found by Kim (1999), Meager *et al.*, (2002), Barlow *et al.* (2004) and Pleasence and Balmer (2012). Individuals also frequently reported their situation or the outcome of the dispute rather than their legal/rights and tended to also provide very vague responses which did not appear to have specific relevance to their particular dispute. In addition, whilst claiming to know their rights, 11.1% went on to claim they ‘didn't know’ in relation to the question. Less frequently, 2.6% of respondents gave verbatim responses which implied an over/under assessment of their rights. Results did not suggest any connection between interpretation of or response to the verbatim question and level of educational attainment.

### Who knows what and when?

In keeping with our first hypothesis, most individuals did not have knowledge of their rights to begin with. These findings are not surprising and are in line with the findings of previous studies, notably that of Buck *et al.*, (2008), Denvir *et al.*, (2012), Parle/IARS (2009) and Casebourne *et al.* (2006). As also hypothesised, the majority (60.7%) of respondents who reported a lack of knowledge at the outset of the problem did not go on to acquire knowledge of their rights to the standard of ‘completely’ or ‘mostly'.

As found by Kim (1999) and Meager *et al.*, (2002) and in keeping with our second hypothesis, we found that the rate of existing knowledge and acquisition of knowledge varied by problem type. Interestingly, many of the problem types for which knowledge was poor at the outset, were related to social exclusion and vulnerability, including rented housing, money, debt, benefits, education and domestic violence as has been noted by both Buck *et al.* (2005, 2008), Balmer *et al.*, (2010) and Denvir *et al.*, (2012). This may be related to the complexity of the problem, or potentially the low rate at which individuals with these problem types seek professional advice (see further Pleasence *et al.*, 2011, p. 43). This would also explain why individuals with divorce and owned housing problems were more likely to acquire knowledge of their rights since these are problem types for which the obtaining of professional advice is far more common (Pleasence *et al.*'s 2011, p. 43).

In keeping with our third hypothesis, based on Deming's (1950) and Groves *et al.*'s (2009) concerns regarding issues of memory recall in relation to self-assessed questions, we found that those who self-reported knowing their rights in Wave 1 of the CSJPS in respect of a particular problem, frequently answered inconsistently when asked whether they knew their rights in Wave 2 (in relation to the same problem). However, more often people professed to having the same if not greater levels of knowledge in relation to their problem in Wave 2. That reductions in levels of knowledge happened more frequently for rented housing, money and personal injury problems may be a reflection of the complexity of dealing with such issues or the fact that individuals tended to overestimate their knowledge of rights in relation to these problem-types more frequently, only realising later down the line. This was certainly the case for those with rented housing problems where 4.6% of verbatim responses were categorised as ‘(3) Potential Error', the third highest of any other problem group. Interestingly, those with money problems tended towards answering the verbatim question by explaining their ‘(1) situation/outcome’ than those with other problem types and those with personal injury problems tended to report their rights as ‘(4) Value judgements’ than other problem types. The fact that they did not answer the verbatim question in the expected manner and the fact that they answered in it a way that suggested they lacked knowledge of their rights, may account for why by Wave 2 they had lowered their self-reported levels of knowledge. Whilst these results highlight how self-assessed knowledge changes over time, neither comparison between waves nor alteration of our question (to ask participants about both their existing and acquired knowledge) can wholly negate the continued issue of memory-recall noted by Deming (1950) and Groves *et al.*, (2009).

Consistent with our fourth hypothesis that many of the people who profess to have knowledge of rights will be unable to articulate these rights in response to the open-ended format, we found that 11.1% of respondents were not able to articulate their rights/legal position when asked to do so. However, a number of other verbatim response types suggested that individuals had a lack of understanding of their rights. We would contend that this was the case in respect of those 58% of respondents who when asked to explain their rights/legal position instead gave a summary of their problem or its outcome, tended to over- or under-estimate their rights, gave a value judgement, mentioned non-specific rights or claimed to have no rights. Yet, although it suggests as much, we cannot conclusively state (contrary to their self-reporting) that these individuals did not know their specific rights in relation to the problem. Whilst on the face of it such responses imply if not a lack of knowledge of rights/legal position, a lack of understanding of what is meant by the term ‘rights’/‘legal position’, these responses may simply be indicative of participants interpreting the question differently than anticipated although we would not expect this to be the case for the entire 58% of respondents who did not answer the open-ended question as anticipated. What we did not find was any evidence to support Geer's (1988) assertion that response types would vary by education level. Although verbatim responses did vary, it did not appear that education was associated with any clear pattern of response or non-response as Geer (1988) suggests.

### What do open-ended questions tell us?

Our results support the proposition that an open-ended question approach to exploring knowledge of rights yields insight into knowledge not acquired by other formats. Unlike hypothetical questions, asking individuals to describe their rights/legal position in their own words gives insight into the knowledge driving their resolution of the problem. The fact that individuals who answered the fixed-choice self-assessment question often then failed to answer the open-ended question (by claiming they ‘don't know’) highlights, as proposed by Schuman and Presser (1981) and Schwarz and Hippler (1991), the tendency of individuals to answer self-assessed/fixed-choice questions with greater confidence/-frequency than open-ended questions. However, the findings detailed above also suggest that the utility of an open-ended question format in the context of a large-scale quantitative survey will remain constrained by difficulty reconciling articulation and interpretation with actual knowledge. Whilst this problem could be avoided through the use of vignettes it would be difficult to do so with real problems. Although the open-ended question enabled individuals to answer freely, as has been previously noted (Geer 1988) it also enabled them to freely interpret the question. There was also some evidence, as Tourangeau and Rasinski (1988) note in relation to fact-based questions, of individuals providing attitudinal responses rather than directly answering the question, as evidenced by those who provided a value judgment/claimed ‘no rights’ in response to the open-ended question. While nearly half of respondents were able to articulate their rights/legal position and interpreted the question as anticipated, there was a limit to the extent to which responses provided clear insight. For example, whilst some individuals referenced the relevant legislation, most did not. Respondents may have been relying on ‘common sense’ when reporting what they believed to be their rights rather than an actual understanding of the law as found by Meager *et al.*, (2002), Barlow *et al.* (2004) and Kim (1999). Conversely, others may have provided an explanation of the situation, even though they did have a clear understanding of their rights. Thus, as found in respect of self-assessed, fact based, and hypothetical questions, an open-ended question is not in itself capable of providing conclusive insight into public knowledge of rights.

### Policy implications

Studies seeking to acquire an understanding of the extent to which the public know their rights, will tend to produce an inflated rate of knowledge where they rely on the public's self-assessment alone. As our results highlight, asking individuals whether they knew or acquired knowledge of their rights yields a greater number of individuals saying yes than those capable of articulating these rights. Similarly, approaches relying on tests of knowledge may just reflect the extent to which respondents guess correctly, rather than reflecting any real knowledge. Including an open-ended question provides some degree of verification, however, in order to determine whether the open-ended responses given derive from knowledge of the law, a further question must be asked, designed to determine how the individual knew their rights. Based on both of the aforementioned considerations, a more nuanced question or series of questions would need to deduce not only what individuals thought their rights to be, but also how they arrived at this conclusion. This may involve a number of prompted open-ended questions that do not fit as readily within a quantitative survey format and may be more appropriate within the context of a qualitative study.

Whilst we would caution against concluding that most people were unable to articulate their rights simply because the answer they provided was not what was anticipated (particularly where this answer fell into the ‘(1) situation/outcome’ category) nonetheless our results suggest some priorities for the development of public legal education. In particular, we note that individuals most often appeared unable to articulate their rights in respect of debt, benefits and education problems. Whilst this may be indicative of the complexity of the problem itself or the capacity of the individual, if we are to assume that knowledge and articulation of knowledge of rights plays a key role in problem resolution, then public legal education may have a role to play in improving knowledge in these areas. In addition, individuals more frequently claimed to have ‘no rights’ in respect of employment and education problems than in respect of other problem types. Rather than a misinterpretation of the question, such responses appear to be indicative of feelings of disempowerment that may stem, not from a lack of knowledge of rights but rather a sense of powerlessness arising from the inequality of the parties. The perceived ‘impotence’ of these rights suggests the need to consider legislative rather than educational intervention/s. In contrast, those reporting ‘vague rights’ (neighbours, owned housing, clinical negligence and domestic violence) may benefit from a more specific understanding of the problem as it relates to their rights. The same is also true of consumer, employment, education and rented housing disputes, where over/underestimation of rights occurred most frequently.

When considering family issues (divorce/relationship breakdown and domestic violence) in comparison to the remaining civil justice issues, individuals did tend to provide verbatim responses which were indicative of the fact that they knew and could articulate their rights. If this (and the more frequent acquisition of knowledge for family law problems) is related to their obtaining of professional advice as noted above, any reduction in the availability of advice is likely to have an impact on the extent to which those with family law problems report knowing their rights in the future. Given that family law is one area where legal aid is set to be substantially curtailed, it suggests an ongoing and long-term role for PLE in buffering the impact of legal aid reform.

Yet in spite of what appears to be continued need for PLE and whilst simultaneously promoting self-help for the range of problems soon to be out of the scope of legal aid, the Government has imposed upon itself no duty to promote knowledge of rights, develop just-in-time legal information, share the third sector's burden of equipping citizens to better handle their problems alone or for that matter, inform itself as to the need for public legal education interventions (see further Legal Aid, Sentencing and Punishment of Offenders Act 2012, s 1(3)^15^). Whilst an expectation that the public takes greater responsibility for their legal problems should be complimented with the tools to enable them to do so, thus far it remains to be seen who will take responsibility for both the research underpinning and the development of PLE.

## Future research

In this study we have used the articulation of rights as a proxy for actual knowledge. Although we recognise that during the course of a dispute some individuals will never have to put into ‘legal terms’ the rights they feel have been infringed, our results provide new insight into how the public articulate their rights and whether open-ended questions might produce more reliable data. Nonetheless, further research utilising different methodologies is needed in order to more reliably determine how well individuals know their rights and, crucially, the role knowledge plays in the resolution of civil and social justice problems. Our assumption remains that knowledge of rights leads to ‘better’ problem-resolution, but there is little reliable evidence to support this assumption and continued difficulty in measuring outcomes. If we are to assume that individuals will in future be required to handle more of their civil and social justice problems alone, it is clear that PLE will continue to have a role to play in access to justice. But whether this role should be to improve knowledge, build confidence, promote early action or a combination of these objectives, cannot be fully understood without further enquiry.

## Notes

1. Other key rationales in determining what would and would not remain in scope, included:

i. The importance of the issue (including personal choices)
ii. The litigant's ability to present their own case (including the venue before which the case is heard, the likely vulnerability of the litigant and the complexity of the law)
iii. The availability of alternative sources of funding
iv. The availability of alternative routes to resolving the issue
v. The need to fulfil our domestic and international legal obligations (including those under the European Convention on Human Rights).

2. Civil Justice problems can also be referred to as ‘justiciable’, ‘legal-related’ or ‘rights’ problems, or ‘problems with a legal dimension’. They are everyday and common-place problems that may or may not be resolved through the civil justice system. Examples of such problems are listed in Table 1.

3. PLE has been used to refer to both ‘legal rights-based education’ intended to promote general public awareness of the law and legal system and ‘self-help/just-in-time’ initiatives aimed at guiding people through the resolution of a particular problem and the legal rights it engages (Giddings and Robertson 2003; Lawler *et al*, 2009). Rights-based education has included the introduction of compulsory citizenship classes within the school curriculum since 2005 and Ministry of Justice (MOJ) support of Public Legal Education (PLE) initiatives through the part funding of the Public Legal Education Network (PLEnet) (PLEAS Taskforce 2007). Efforts to provide self-help/just-in-time resources have not been centrally coordinated, but have included the publication of a number of information leaflets available online and in person from a range of agencies including public and third sector organisations (e.g. Shelter, Citizens Advice, Money Advice Trust and government affiliated websites, including the Office of Fair Trading and the now defunct Community Legal Advice, DirectGov and National Debtline to name but a few).

4. This was particularly so in respect of discrimination-like actions which at the time did not fall under discrimination legislation, but which would have fallen under unfair dismissal, including dismissal on the grounds of sexual orientation and age.

5. For policy makers, this is perhaps not a pressing concern where ethical/social norms continue to align, but does suggest that where the law changes, those people deemed to ‘have knowledge’ may not necessarily hold contemporaneous views any more.

6. In addition, one wonders whether the questions asked of Kim's (1999) respondents were those which individuals could reasonably have been expected to know. An example included a question which asked whether it was lawful to fire an employee as retaliation for refusing to participate in an illegal billing practice. Whilst the practice was lawful in New York (as opposed to the other two states where the study took place), for New York respondents to have known this would have required a detailed understanding of appeal court case law (Kim 1999, p. 469). Knowing the law in respect of rather obscure (and less likely) dismissal-at-will circumstances is arguably less of an imperative than providing a correct gauge of public knowledge relating to more likely dismissal-at-will events.

7. These situations included a woman's request for financial support post-relationship breakdown, a woman's entitlement to inheritance upon the death of her partner and an unmarried father's right to make decisions regarding medical treatment of his biological child vies-a-vie his rights as a married man.

8. The idea that couples accrued greater rights as time went on.

9. The CSJPS is a substantially developed form of Genn's (1999) Landmark ‘Paths to Justice’ Survey.

10. So, where an individual provided a response that could have been coded as (1) or (4) for example in relation to an employment problem they said ‘I was in the right, I had been a good employee for over four years and I was then fired’ they were coded as (1) because their explanation of the outcome of the case was longer than their (4) value judgement.

11. For nine problems respondents refused to give an answer (0.5%).

12. We also fitted two binary logistic regression models in order to predict the likelihood of respondents reporting knowledge of their rights from the outset (Model 1) or later gaining knowledge of their rights (Model 2) by problem-type whilst controlling for a range of socio-demographic characteristics (age, academic qualifications, gender, tenure, family type, illness/disability, mental health, ethnicity and income). Results yielded similar findings to those detailed in Table 2. Readers wishing to know more about these models should contact the authors.

13. For purchases made in England and Wales, with purchases from sellers within the EU protected by the European Union Distance Selling Directive 97/7/EC.

14. Even when splitting education level into a binary ‘education qualification’ and ‘no educational qualifications’ category, no significant pattern emerged to suggest that education level had any role to play in type of verbatim response given.

15. Note in particular, the wording used in Section 1(3) of the Legal Aid, Sentencing and Punishment of Offenders Act 2012, where it says ‘The Lord Chancellor **may** secure the provision of — *(a) general information about the law and the legal system, and (b) information about the availability of advice about, and assistance in connection with, the law and the legal system.*
